# A Low-Cost LED-LDR portable colorimeter-multimeter (LED-LDR PCM) for analytical laboratories in Iraq: An educational of deviation from the beer-lambert law and green chemistry approach

**DOI:** 10.1016/j.mex.2026.103881

**Published:** 2026-03-24

**Authors:** Ali Amer Waheb, Ruba Fahmi Abbas, Mohammed Jasim M. Hassan, Dhifaf A. Abdulabbas, Fatimah A. Abed

**Affiliations:** a Chemistry Department, College of Science, Mustansiriyah University, Baghdad, Iraq; b Polymer Research Unit, College of Science, Mustansiriyah University, Baghdad, Iraq

**Keywords:** Educational LED-LDR, Methyl red, MoGAPI, Greenness and whiteness assessment, CaFRI

## Abstract

A new, low-cost LED-LDR Portable Colorimeter-Multimeter (LED-LDR PCM) was developed as an educational tool for undergraduate chemistry students. This device constructed from inexpensive and available components in the Iraqi markets. These included a multimeter, a white LED light source, a light dependent resistor (LDR), a battery, and a sample cell (cuvette). This device serves as platform for teaching essential principles of the systematic deviations from the Beer-Lambert law with polychromatic LED sources, build calibration curves, and teaching self-filtering effect phenomenon. The LED-LDR PCM device showed a high accuracy and precision in the determination of methyl red dye in tap water samples, with RSD% values from 0.791% to 3.076% and recovery values ranging from 99.131% to 101.562%. The performance of the educational LED-LDR PCM device was evaluated using greenness and Whiteness assessment tools, such as GAPI, MoGAPI, CaFRI, and the Multi-Color Assessment (MA) Tool. A comparative analysis against reported methods revealed that the educational LED-LDR PCM device is more sustainable and environmentally friendly, with a high overall Whiteness score of 66.9%, showed the scores of reported methods. This project highlights a new direction for the development of simple, cost-effective analytical devices that are ideal for educational purposes analytical devices and aligned with the principles of green analytical chemistry.

## Graphical abstract

**Figure:**
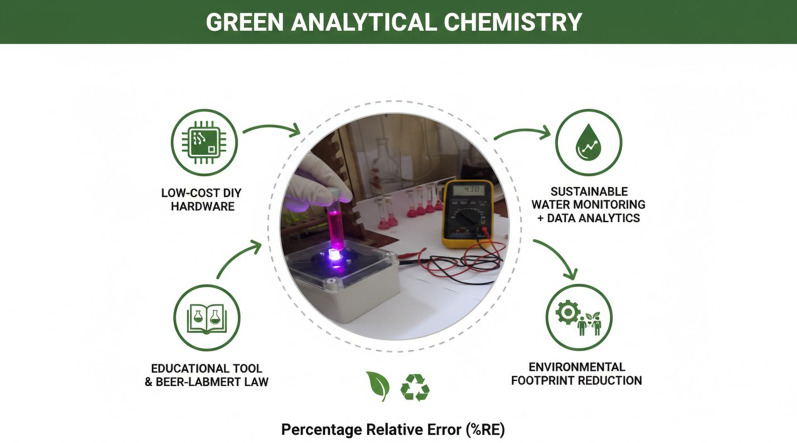

Specifications table

**Table:** Specifications table.

**Subject area**	Chemistry
**More specific subject area**	Analytical Chemistry / Green Chemistry
**Name of your method**	LED-LDR Portable Colorimeter-Multimeter (PCM) method
**Name and reference of original method**	The method is based on the fundamental principles of the Beer-Lambert Law. It is a customized instrumentation developed by the authors to adapt standard spectrophotometric principles into a low-cost, portable LED-LDR system for educational tool for undergraduate chemistry students.
**Resource availability**	The components used in this method are globally available, low-cost, and can be easily sourced from electronic commercial markets. The system comprises a standard 5 mm Light Emitting Diode (LED) as the radiation source and a Photoresistor (LDR) as the detector. Power is supplied by a rechargeable 3.7 V Lithium-ion battery (compatible with a Durapro LCD USB charger for sustainability). A Digital Multimeter is utilized for signal readout, and the entire assembly is housed in a lightweight, durable plastic body to ensure portability and protection of the optical path.

## Background

The fundamental linear relationship between the absorbance of analyte and the concentration of the same analyte called Beer-Lambert Law and leaching this law is important for undergraduate chemistry students [1]. There are many chemical and instrumental deviations of the Beer-Lambert Law must be students recognize and learning them. Many last reported studies in many fields such as microbiology, physics, and atmospheric [2]. Chemical analysis has attended a growing trend toward on-site (in situ) or field laboratory testing. These miniaturized technologies have contributed to the development of new green teaching methodologies and innovative applications [3]. A locally made, low-cost chemical analysis device shows low manufacturing and operating costs compared to specialized commercial devices [4]. Such locally manufactured devices can be produced at a significantly lower cost by relying on simple, affordable components such as light-emitting diodes (LEDs), voltmeters, and photoresistors. Being battery-powered, the device also consumes less energy and does not require expensive chemicals or costly maintenance [5]. This locally made device avoids sample transport, reduces analysis time, and eliminates the need for preservatives. Analysis can be performed directly on-site, negating the necessity of transporting samples to a laboratory and thus reducing the probability of sample change or damage due to environmental conditions such as effect of light, temperature, time delays [6,7]. These minimize devices teaching students the environmental sustainability is achieved through reduced energy consumption due to its small battery, which contrasts with larger devices requiring a constant electrical current. Additionally, on-site analysis contributes to lower chemical consumption by eliminating the need for sample preservation and transport chemicals, consequently reducing chemical waste associated with laboratory analyses. The device's design aligns with the principles of green chemistry, aiming to minimize the environmental impact of chemical processes. The locally manufactured device has diverse applications, including environmental analyses (such as water, soil, and air analysis in various locations), agricultural analyses (such as soil and plant analysis to determine necessary nutrients and fertilizers), and educational analyses, making it suitable for teaching students the principles of chemical analysis in a practical and simplified manner in schools and universities [[8], [9], [10], [11], [12]].

Determining the concentration of dissolved substances by spectrophotometry using the Beer-Lambert law is an important component of chemistry curricula worldwide. However, access to modern laboratory equipment, especially spectrophotometers, is sometimes limited for students to conduct analytical experiments. Previous research on the use of light-emitting diodes (LEDs) for analytical chemistry activities began shortly after LEDs became widely available in the 1970s based on VOSviewer software and literature from Scopus databases to create maps of bibliometric network. The accessibility of low-cost microcomputers also led to numerous reports of multifunctional homemade spectrophotometers. However, most of these require some knowledge of programming and electronic circuit design [11]. In designing the colorimeter, several detectors were employed. LED-LDR based colorimeter was used for determination of Cu in copper in urine and water [13], a green-color LED (520–620 nm)-photoresistor was used to determination of eriochrome black T, permanganate, and Fe(II)-1,10-phenanthroline complex [14], LED-photoresistor was used to estimate of carmoisine and ponceau dyes [15], determination of N and P in soil [16], IR-LED-phototransistor (890 nm) determination As in water [17], LED-phototransistor-based on ARS-Zr dyes as the sensor reagent to estimate of F in water [18], LED (red-green-blue) with two photodiodes as detector was used to determination of NO₂⁻ and Fe in river [19], LED (red-green-blue) was used to determination of Fe, Cu, and, Cr [20], planar-type silicon photoelectric detector to determination of glucosein water [21], LED- silicon photodiodes was used to determination of F- in drinking water [22], LED (360 nm)-silicon photodiode was used for determination of fluorescent bleaching agents in paper [23], LED- silicon photodiodes was used to determination of phosphates in water agricultural samples and salicylic acid in an acne medication [24], the light source LED (355 nm)-another LED (355 nm) as the detector was used to determination of folic acid and cinnamaldehyde [25], LEDs as both the light source and detector was used to determination of Fe in water [26].

The polychromatic sources are the most problem in the Portable Colorimeter-Multimeter due to deviation of the Beer-Lambert law and lead to absorbance offsets. the Beer-Lambert law is (*A*=ϵbc) which used with monochromatic light LEDs, but when it used white LEDs with a broad spectral bandwidth leads to non-linear systematic error [27]. The Beer-Lambert law deviation can used as in an educational study and it allows students to study these deviations as an exercise in error analysis.

**Fig. 1:**
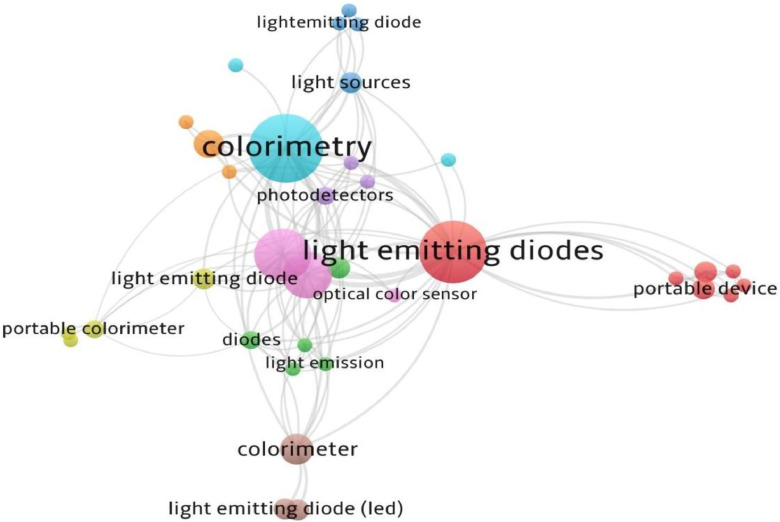
The bibliometric network of different LED-LDRs used for as colorimeter [12,28,[13], [14], [15], [16], [17], [18], [19], [20], [21], [22], [23], [24]].

The aim of this study is to develop a simple, portable, and low-cost colorimeter for educational case of measure the concentration of Methyl red dye and it which making to close the gap between theoretical learning and real application of the Beer-Lambert law deviation. The device is constructed using readily available and inexpensive components from local markets in Iraq, including a white LED light source, a light-dependent photoresistor (LDR), a multimeter, and a rechargeable battery (LED-LDR PCM). The LED-LDR PCM device provides an easy and direct method for estimating solution concentration by measuring the change in electrical resistance caused by light absorption, thereby offering a practical and environmentally friendly alternative to more complex and expensive instruments. The results compared to a conventional UV–Vis spectrophotometer. This locally made LED-LDR PCM device is an educational tool, allowing chemistry students to understand experience have reinforcing key analytical principles such as the Beer-Lambert law and colorimetric analysis. It serves as an ideal, low-cost alternative for educational institutions with limited budgets. Furthermore, the study evaluates the environmental sustainability of the new method using GAPI, MoGAPI, CaFRI, and Multi-Color Assessment (MA) tools, showing it to be a greener alternative to traditional analytical techniques (Fig. 1).

## Method details

### Design of the LED-LDR portable colorimeter-multimeter (LED-LDR PCM)

In this study, the use the light source of a white LED, rather than a monochromatic LED or a specific color filter, was a studied design choice to create a simple and low-cost educational tool. A white LED is inexpensive and widely available in Iraqi local markets, which is important for use in educational analytical chemistry labs that limited budgets. The . 2, . 3, . 4, . 5, . 6, . 7 depict images of the locally manufactured (LED-LDR PCM) device. It is constructed from locally sourced plastic with a transparent outer cover into which the solution cell is inserted. The black plastic body houses:

**. 2:**
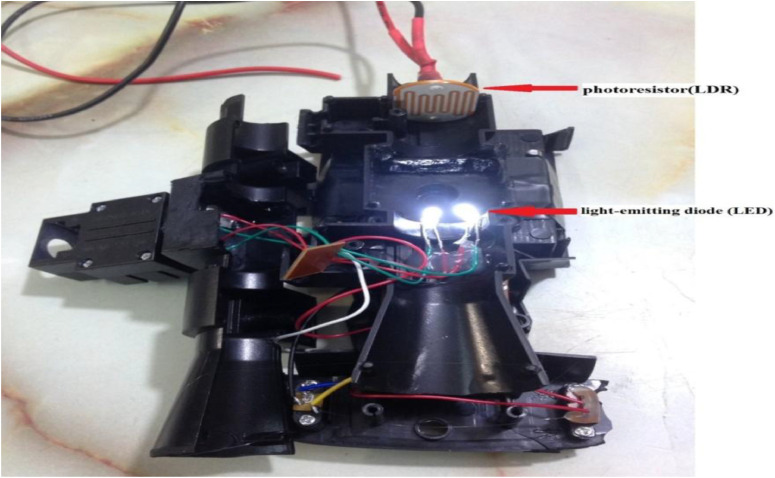
Internal view of the black plastic body containing the white light source (LED) and the photoresistor (LDR).

**. 3:**
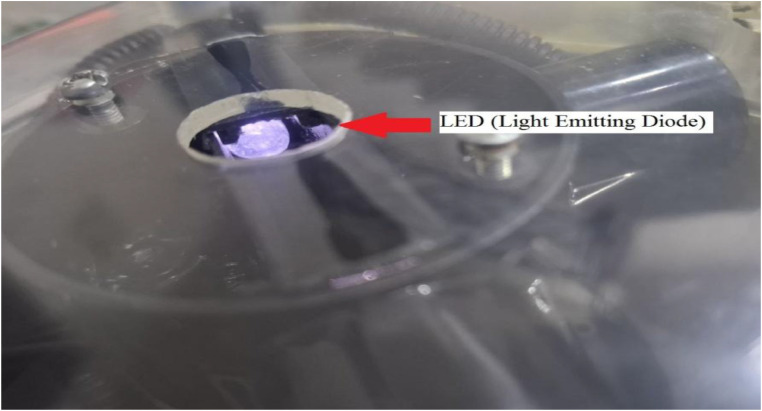
Internal view of the white light source (LED).

**. 4:**
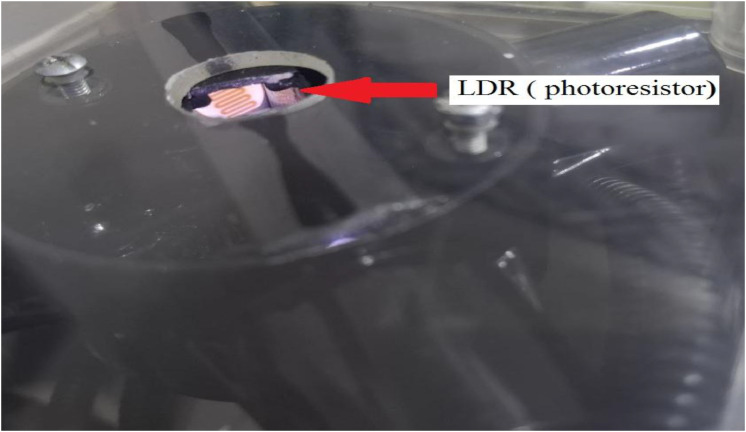
Internal view of the photoresistor (LDR).

**. 5:**
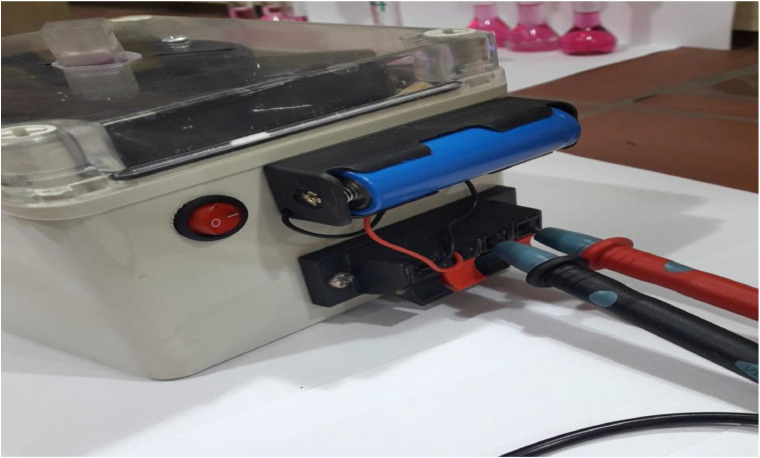
External view of the black body placed inside another plastic box and externally connected to a battery and a multimeter.

**. 6:**
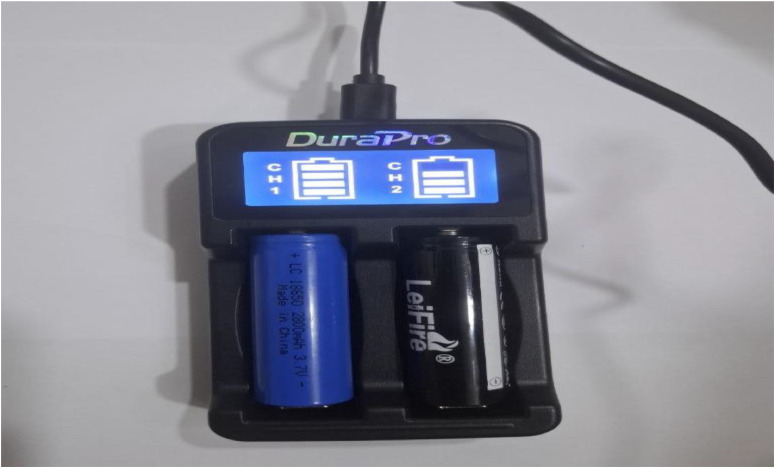
Image showing lithium battery being charged by an external charger; the battery offers 48 h of operation without charging.

**. 7:**
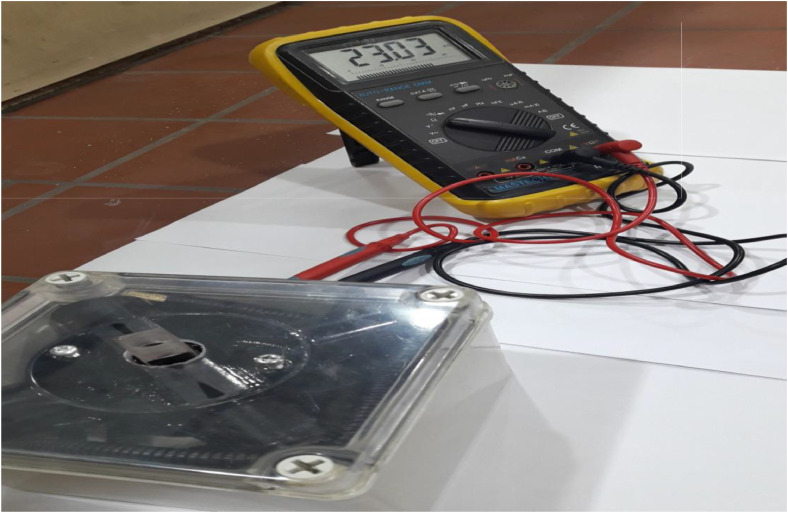
Front view of the complete LED-LDR PCM device with sample cell.

• A white light source (LED) (5 mm White LED, Optoelectronic Co., Ltd., China). The white LED emits a continuous spectrum of visible colors ranging from 400 to 800 nm.
• A light-dependent photoresistor (GL5506 LDR).
• The power source for the device is a 3.7 V rechargeable lithium-ion battery with dimensions of 18 mm x 65 mm. This battery is charged using an external charger and can operate for 48 h without needing to be recharged.
• A digital multimeter, a versatile electronic measuring device, is used to measure various electrical properties in electronic circuits. In this device, it measures electrical resistance, which is quantified in ohms (Ω) and represents a material's opposition to the flow of electric current.
• A sample cell (a cuvette with a path length of 5 mm) is utilized.
• The battery is connected to the multimeter on one side and to the LED light source on the other. Light emitted from the LED passes through the solution cell, and the resulting change in resistance, due to the colored solution absorbing the transmitted light, is measured by the multimeter.
• Table 1 presents the approximate cost of the device's components, totaling approximately $24, which is equivalent to roughly 24 $.

**Table 1:** The price of the device's components includes a voltmeter, LED, and LDR resistor (LED-LDR PCM).

Part	Supplier	Price, $
LED (Light Emitting Diode)	5 mm White LED, Optoelectronic Co., Ltd., China	2 $
LDR (photoresistor)	GL5506 LDR Resistor Light-Dependent, China	2 $
Digital Multimeter	Multimeter digital Mastech 68, China	8 $
Lithium-ion battery with 3.7V	3.7v 280mah battery, China	2 $
Durapro LCD USB	China	6 $
plastic body	Iraq	4 $
**Total**		**24 $**

### Preparation of standard solutions

#### Preparation of a calibration curve for methyl red dye

0.1 g of methyl red dye (C_15_H_15_N_3_O_2_, M.wt of 269.304 g/mol, Sigma-Aldrich, ≥99.0%) was weighed and dissolved in a small amount of distilled water. The solution was then transferred to a 100 ml volumetric flask and diluted to the mark with distilled water to prepare a stock solution with a concentration of 1000 mg/L. The following volumes (0.5, 0.75, 1, 1.25, 1.5, 1.75, and 2) ml were accurately measured using a micropipette from the 1000 mg/L stock solution and transferred to separate 5 ml volumetric flasks. Each flask was then diluted to the mark with distilled water to obtain standard solutions with concentrations of (100, 150, 200, 250, 300, 350, and 400) mg/L [29,30].**Figure S1** shows the measurement of methyl red dye at concentrations ranging from 100, 150, 200, 250, 300, 350, and 400 mg/L using the new locally (LED-LDR PCM) device. The analytical performance of the developed LED-LDR PCM device was validated according to the ICH Q2 (R1) guidelines (covering linearity, accuracy, precision, and detection limits) [31,32].

### Spectrophotometric method

To compare with the proposed method and demonstrate its validity, the standard solutions prepared above were measured spectrophotometrically after the spectrophotometer was calibrated using distilled water.

## Method validation

In this study, a simple colorimeter educational device for measuring colored solutions was constructed from inexpensive and readily available components in the Iraqi markets. These included a multimeter, a white LED light source, a light dependent resistor (LDR), a battery, and a sample cell (cuvette). This device measures the light incident on the colored solution in ohms, offering an easy and direct method for estimating the solution's concentration. The new LED-LDR PCM device is easy to carry and use, energy-efficient, and utilizes a colorimetric method, making it an ideal choice for many applications and is also environmentally friendly. The principle of operation for this device is that a photocell converts light into an electrical current. When light falls on the photocell, it produces a current proportional to the amount of incident light. The dye whose concentration is to be measured is placed in the light path falling on the photocell. The dye absorbs a portion of the light, thereby reducing the electrical current produced by the cell (i.e., increasing its resistance). Finally, the multimeter measures the change in electrical current (or resistance) resulting from the dye's absorption of light. The resistance (R) is inversely proportional to light transmittance (T) and absorbance (A) can calculate by using the modified equation of the Beer–Lambert. The negative mark will be changed to a positive by using this modified law. So, resistance is converted to absorbance using this law [33]:

$$\mathrm{Absorbance}=\;-\;\log\;\left(\frac{T_{\mathrm{sample}}}{T_{\mathrm{blank}}}\right)=\;\log\;\left(\frac{R_{\mathrm{sample}}}{R_{\mathrm{blanck}}}\right)$$

Resistance readings were recorded in kilohms (KΩ) and converted to absorbance, as shown in Table **S1**.

**Fig. 8:**
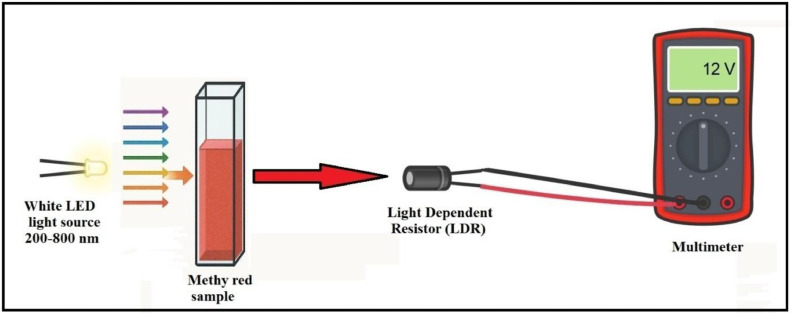
Schematic Diagram of LED-LDR PCM device.

In this study, we used Methyl red as sample. When white light falls on a red-colored solution, the solution will absorb the complementary colors to red. This means it will absorb most of the green and blue wavelengths from the visible spectrum. So, a red solution appears red because it primarily reflects or transmits red light while absorbing other colors (Fig. 8).

## Calibration curve

In this study, the introduce students to the limitations of optical measurements could be present a good educational by studying the compare between the educational LED-LDR PCM results and the reference UV–Vis instrument, and let students to learn of formal errors coming from optical design instead of using "black box" inside of the traditional instrument.

Table 2 shows the absorbance values calculated from the resistance using the new educational LED-LDR PCM device and a UV–Vis spectrophotometer for a range of concentrations from 100 mg/L to 400 mg/L of standard methyl red solution. the Percentage Relative Error (RE%) or percentage deviation (%) from the Beer-Lambert Law calculated for each concentration point to obtain the quantify measurement of the instrumental deviation, as following equation [34]:

$$\mathrm{Percentage}\;\mathrm{Relative}\;\mathrm{Error}\;\left(\mathrm{RE}\%\right)\;=\frac{Abs_{\left(\text{LED}-\text{LDR}\;\text{PCM}\right)}-Abs_{\left(\text{UV}-\text{Vis}\right)}}{Abs_{\left(\text{UV}-\text{Vis}\right)}}\times 100$$

**Table 2:** Calculated absorbance values for standard methyl red solutions obtained from an educational portable colorimeter (based on resistance change measurement and LED light source) (LED-LDR PCM) compared to absorbance values obtained from a UV–Vis spectrophotometer.

Dye Concentration (mg/L)	Absorbance of educational LED-LDR PCM	Absorbance of UV−vis Spectrophotometer at 523 nm	Percentage Relative Error (RE%)
100	0.862	0.066	1206.060
150	0.890	0.180	394.444
200	0.924	0.271	240.959
250	0.955	0.385	148.051
300	0.988	0.481	105.405
350	1.020	0.577	76.776
400	1.053	0.666	53.722

It was observed that at low concentration (100 mg/L) the value of **RE%** became higher (1200%), but the value of **RE%** became lower (52.8%) at higher concentrations (400 mg/L). This is because the LDR saturated with non-absorbed wavelengths from methyl red solution (emitted from white LED) at lower concentration (Table 2). As concentration increases, the sample acts as a selective spectral filter, absorbing these interfering wavelengths and narrowing the effective bandwidth. This 'self-filtering' effect reduces the analytical offset, causing the response to converge toward the monochromatic UV–vis reference values.

At higher concentration, the methyl red sample absorbing interfering wavelengths, act as a selective spectral filter (called self-filtering) making to reduce the analytical **RE%**, and becoming closer to the absorbances values of monochromatic UV–vis reference [35].

In Table 3, the results showed a good correlation coefficient (R²) 0.999 for educational LED-LDR PCM. The LOD was 20.808 mg/L and the LOQ 63.057 mg/L, which indicates a good ability for determining Methyl Red dye.

**Table 3:** Validation parameters of educational LED-LDR PCM and UV−vis Spectrophotometer at 523 nm for determination of methyl red dye.

Method	Calibration equation	Linearity range (mg/L)	Correlation coefficient (R^2^)	Accuracy (mean±SD)	RSD%	LOD (mg/L)	LOQ (mg/L)
**Calibration curve of educational LED-LDR PCM**	*Y* = 6.40 × 10^–4^*X* + 0.795	100–400	0.999	103.517±1.755	1.695	20.808	63.057
**Calibration curve of UV−vis** **Spectrophotometer at 523 nm**	*Y* = 0.002X-0.133	100–400	0.999	102.083±1.653	1.619	27.066	82.018

A calibration curve created with a high concentration range (100- 400 mg/L) will inherently yield higher LOD and LOQ values compared to a curve calibrated for a lower concentration range due to these LOD and LOQ are statistically calculated from the standard deviation of the regression line's response. This is a normal and expected result and does not necessarily indicate a poor performance of the portable colorimeter LED-LDR PCM device, but its intended purpose of measuring high-concentration solutions.

The student can learn the calculating of the sensitivity ratio from the slope in calibration curves as following:

$$\text{sensitivity}\;\text{ratio}=\frac{slope_{\left(\text{LED}-\text{LDR}\;\text{PCM}\right)}}{slope_{\left(\text{UV}-\text{Vis}\right)}}=\frac{0.002}{6.40\;\times \;10{\;}^{-4}}=3.12$$

This value is mean that the UV–Vis spectrophotometer device is 3 times more sensitive than the educational LED-LDR PCM device, and this is due to the use of monochromatic light, which increases absorption efficiency. The maximum absorption efficiency for methyl orange dye (λ_max_)​ and the high spectral purity in UV–Vis spectrophotometer because of using the tungsten-halogen lamp coupled with a diffraction grating. So, the slope of the response increased to 0.002. On the other hand, the broad background illumination introduced in white LED source led to non-absorbed wavelengths reach the LDR detector and reducing the slope of educational LED-LDR PCM device to 6.40 × 10^–4^.

To assess the accuracy of the educational LED-LDR PCM device, the same solutions were measured using a UV–Vis spectrophotometer at a wavelength of 523 nm. These values represent the reference measurements against which the results will be compared. The results shown in Table 4 indicate that the proposed method has A good accuracy and precision, the recovery (Rec.%) being 103.555, 100.740, and 98.412% for three different concentrations of Methyl red dye (intra-day), and a good RSD% of 1.684, 1.620, and 2.664%.

**Table 4:** Accuracy and precision of portable colorimeter (LED-LDR PCM) for the determination of Methyl red dye.

Method	Taken(mg/L)	Found*(mg/L)	Error* %	RSD*%	Rec.*%	Recovery absolute error (%)
**LED-LDR PCM**	150	151.111	0.740	1.684	100.740	−2.463
	250	258.888	3.555	1.620	103.555	2.170
	350	344.444	−1.587	2.664	98.412	−1.717
**UV−vis** **Spectrophotometer at 523 nm**	150	154.805	3.203	1.474	103.203	
	250	253.463	1.385	2.045	101.385	
	350	350.453	0.129	2.569	100.129	

⁎ *n* = 3.

The student can learn the calculating of the recovery absolute error (%) from the recovery percentage (Rec.*%) for both of an educational (LED-LDR PCM) (as measured value) and a UV–Vis spectrophotometer devices (as true or accepted value) in accuracy and precision table as following:

$$\text{Recovery}\;\text{absolute}\;\text{error}\;\left(\%\right)\;=\text{Rec}.{\%}_{\left(\text{LED}-\text{LDR}\;\text{PCM}\right)}-\text{Rec}.{\%}_{\left(\text{UV}-\text{Vis}\right)}$$

The small Recovery absolute error (%) values found to be (−2.463, 2.170, and −1.717) indicate the fact that the final results (concentration and recovery) are highly accurate even the deviation in the absorption values (1200% at 100 mg/L in Table 2 due to white light) and that teaching the student that suitable calibration curve can eliminate minor device defects.

**Fig. 9:**
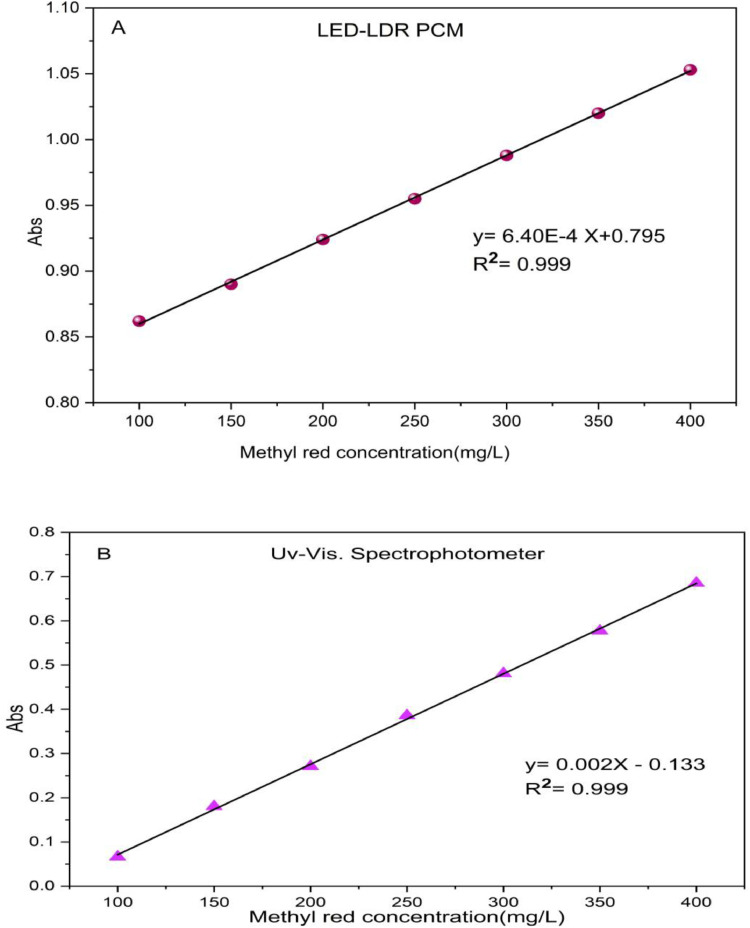
Calibration curve for standard solutions obtained from a portable colorimeter based on measuring the change in resistance and LED as a light source (LED-LDR PCM), and compared to the calibration curve obtained from a UV-visible spectrometer as reference device.

Fig. 9 displays calibration curves for the same standard methyl red solution by using a portable colorimeter that depend on measuring changes in resistance and uses an LED as its light source and compared with a UV–Vis spectrophotometer as reference device.

## Application in tap water samples

For educational purposes, this application provides students with a practical way to determine methyl red dye levels. An educational portable colorimeter (LED-LDR PCM) was successfully used for this purpose on tap water samples collected from Palestine Street and Hay al-Binook in Al-Rusafa, Baghdad, Iraq. Table 5 presents the results for the determination of methyl red dye in 5 mL tap water samples, which were spiked at concentrations of 200 mg/L and 300 mg/L (*n* = 3). The analysis yielded recovery values ranging from 99.131% to 101.562% and RSD% values from 0.792% to 3.076%. These results indicate the new LED-LDR PCM has a high accuracy and precision. A low Recovery absolute error (%) values (−0.809, −2.048, −0.651, and −0.687) in the application table teaching students that the educational (LED-LDR PCM) is highly reliable for field applications comparable to complex devices as UV–Vis spectrophotometer in field quantitative analysis.

**Table 5:** Determination of Methyl red dye using an educational LED-LDR PCM **in tap water samples.**

Water samples	Concentration of Methyl red (mg/L)	LED-LDR PCM			UV−vis Spectrophotometer at 523 nm			
	Add	Found± SD*	RSD*%	Rec.%	Found± SD*	RSD*%	Rec.%	Recovery absolute error (%)
**Location1(Palestine Street)**	200	201.473 ± 1.974	1.959	100.736	203.900 ± 1.191	1.173	101.545	−0.809
	300	297.395 ± 2.168	2.187	99.131	303.538 ± 0.641	0.633	101.179	−2.048
**Location2 (Hay al-Binook)**	200	203.125 ± 3.125	3.076	101.562	201.823 ± 0.609	0.603	100.911	−0.651
	300	301.041 ± 0.795	0.792	100.347	303.103 ± 1.237	1.224	101.034	−0.687

⁎ *n* = 3.

## Evaluation greenness and whiteness assessment tools for a new LED-LDR PCM device

The aim of this research is to design and evaluate a portable colorimeter built from simple, low-cost components for on-site chemical analysis of methyl red dye. This device also serves a crucial educational purpose, providing a hands-on platform for teaching students the fundamental principles of colorimetry and the Beer-Lambert law. Sustainability is a fundamental part of modern scientific research, and teaching students the principles of green analytical chemistry is crucial as it prepares them to be environmentally conscious students. This device aligns with the principles of Green Analytical Chemistry by being easy to carry, energy-efficient, and reducing the need for transporting samples to a lab. The colorimeter's design incorporates a multimeter, a white LED as a light source, and a light-dependent photoresistor (LDR) as a detector. The LED-LDR PCM device measures the change in the LDR's resistance as light from the LED passes through a colored solution.

**Fig. 10:**
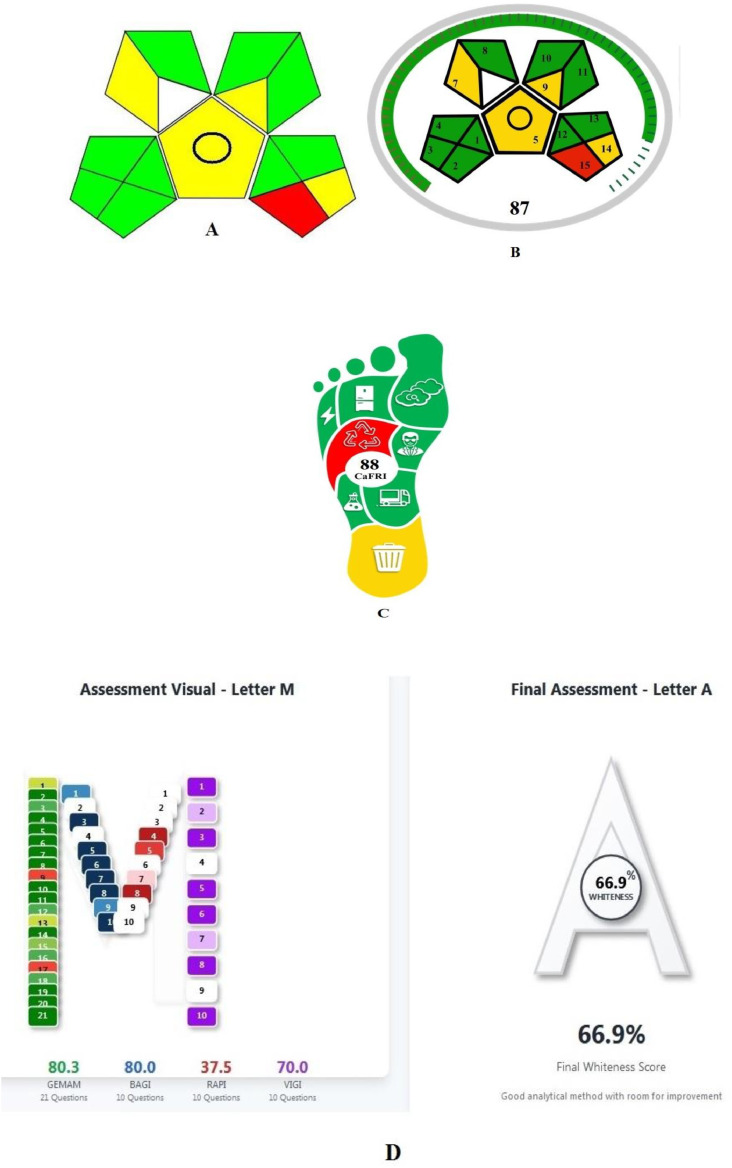
Pictograms of A) GAPI, B) MoGAPI, C) CaFRI, and D) MA tools for Evaluation Greenness and Whiteness Assessment Tools for a new LED-LDR PCM device.

This resistance reading is then converted into absorbance using a modified Beer-Lambert law. The portable colorimeter device was evaluated by J. Płotka-Wasylka using the Green Analytical Procedure Index (GAPI) and Fotouh R. Mansour et al. using the Modified GAPI (MoGAPI) [[36], [37], [38]]. Both tools depend on visual colors to assess the greenness of analytical methods, where a green for more environmentally friendly, yellow for moderate, and red for weak environmental friendliness. On the other hand, the MoGAPI tool adds a scoring system to the assessment, where a score of ≥75 is excellent green, 50–74 is acceptable green, and <50 is not green. In this study, both assessments had 9 green, 4 yellow, and 1 red pictogram, indicating that the analytical method used aligns significantly with the principles of green chemistry. The MoGAPI total score of 87, which is higher than the (excellent green ≥75), further confirms that the portable colorimeter achieved a high degree of compliance with green chemistry principles. The portable colorimeter device was evaluated by Fotouh R. Mansour and Paweł Mateusz Nowak using the Carbon Footprint Reduction Index (CaFRI) [39]. It has been focused on estimating greenhouse gas emissions. This portable device has a high score of 88 on the CaFRI for its low carbon footprint. This is due to a design that minimizes resource and energy consumption by using a small (3.7 V) rechargeable lithium-ion battery. The carbon footprint per full battery charge is estimated at a very low 0.0293 kg of CO_2_, which is a negligible figure compared to conventional lab devices that significantly contribute to Iraq's high carbon emissions. This portable device, with its on-site measurement capability, completely eliminates the need for sample transportation and storage, thereby reducing its carbon footprint. Furthermore, the LED-LDR PCM device uses very little reagent (only 5 ml of a methyl red solution in water) per sample, which minimizes both waste and environmental impact. This device is semiautomated, simple, and single-operator requirement make it efficient**(Table S2)**. The portable colorimeter device was evaluated by Ahmed Emad F. Abbas et al. using the new Multi-Color Assessment (MA) tool [40]. This tool developed 51-question protocol to calculate a whiteness score of analytical method based on four different assessments are: 1-Green Evaluation Metric for Analytical Methods (GEMAM), 2-Blueness Assessment Graphical Index (BAGI), 3-Redness Analytical Performance Index (RAPI), and 4-Violet Innovation Grade Index (VIGI). In this study, the Whiteness Score of 66.9% based on four assessment criteria: 1-GEMAM score of 80.3%, 2- BAGI score of 80.0%, 3-RAPI score of 37.5%, and 4-VIGI score of 70.0%. These scores indicate that the portable colorimeter device is highly practical, environmentally sustainable, and shows a good degree of technological innovation. The low RAPI score was expected due to the inherent limitations of the method's analytical performance, which include a lack of intermediate precision, reproducibility, factor, and interferent tests**(Table S3)**. A comparative analysis of the new LED-LDR PCM device with reported methods is presented in Table 6, with details on the reported methods provided in **Table S4**. The results indicate that the new device is more environmentally useful and friendly. The LED-LDR PCM device has a more favorable "green" pattern than the reported methods in GAPI and MoGAPI tools. Its MoGAPI numerical value of 87 is higher than the values of 66 and 70 for the reported methods. In CaFRI tool, the new device has final score of 88 is significantly higher than the reported methods, which scored 60, 63, and 55. Moreover, in MA tool, the LED-LDR PCM device achieved a Whiteness Score of 66.9%, which exceed a final score of the reported methods (55.3%, 48.6%, and 58.3%) [[41], [42], [43]] (Fig. 10).

**Table 6:** Comparison of a new LED-LDR PCM device with the previously reported methods using GAPI, MoGAPI, CaFRI, and MA tools [[44], [45], [46]].

## Limitations

None.

## Ethics statements

This work did not involve any studies with human participants or animals performed by the author

## Supplementary material *and/or* additional information [OPTIONAL]

Additional supporting data for this method are provided in the supplementary file, including: **(1)** Visual documentation of methyl red analysis (Fig. S1), **(2)** Raw data for resistance-to-absorbance conversion (Table S1), and **(3)** Method sustainability assessment using Green Analytical Chemistry metrics (Tables S2–S4)

## CRediT authorship contribution statement

**Ali Amer Waheb:** Validation, Investigation, Software. **Ruba Fahmi Abbas:** Project administration, Writing – original draft, Writing – review & editing. **Mohammed Jasim M. Hassan:** Methodology. **Dhifaf A. Abdulabbas:** Project administration. **Fatimah A. Abed:** Formal analysis.

## Declaration of competing interest

The authors declare that they have no known competing financial interests or personal relationships that could have appeared to influence the work reported in this paper.

## Data Availability

Data will be made available on request.

## Appendix Supplementary materials

Image, application 1
