# Supplementary material for: A Low-Cost LED-LDR portable colorimeter-multimeter (LED-LDR PCM) for analytical laboratories in Iraq: An educational of deviation from the beer-lambert law and green chemistry approach
Source: MethodsX. 2026 Mar 24;16:103881. doi: 10.1016/j.mex.2026.103881 (PMC13058991; doi:10.1016/j.mex.2026.103881)
Supplement: Supplementary file 1 [file mmc1.docx]

**A Low-Cost LED-LDR Portable Colorimeter-Multimeter (LED-LDR PCM) for Analytical Laboratories in Iraq: An Educational of Deviation from the Beer-Lambert Law and Green Chemistry Approach**

Ali Amer Waheb^1^, Ruba Fahmi Abbas^1*^, Mohammed Jasim M. Hassan^1^, Dhifaf A. Abdulabbas^1^, Fatimah A. Abed ^2^

^1^Chemistry Department, College of Science, Mustansiriyah University, Baghdad, Iraq

^2^Polymer Research Unit, College of Science, Mustansiriyah University, Baghdad, Iraq

Email^*^: [**rubaf1983@uomustansiriyah.edu.iq**](mailto:rubaf1983@uomustansiriyah.edu.iq)

| 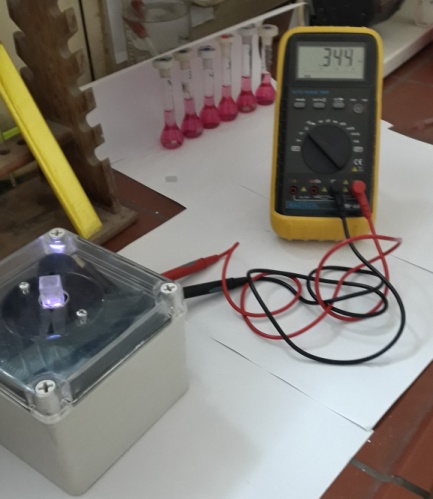  100 mg/L | 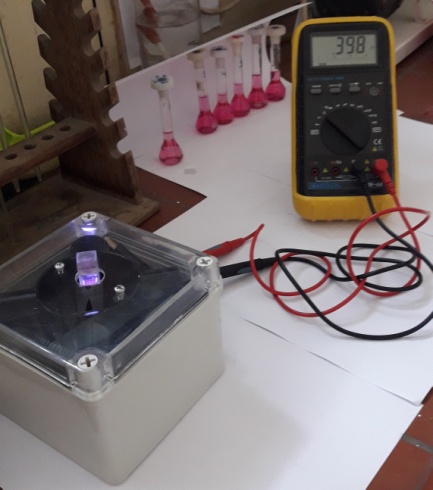  150 mg/L |  |
| --- | --- | --- |
| 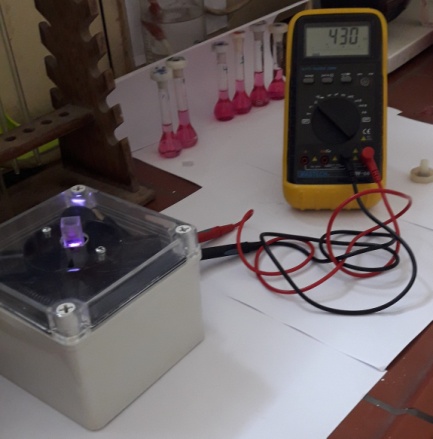  200mg/L | 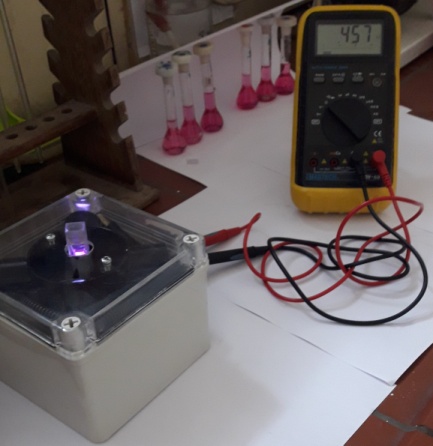  250 mg/L |  |
| 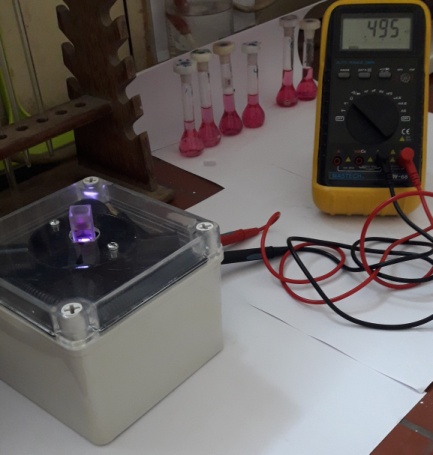  300mg/L | 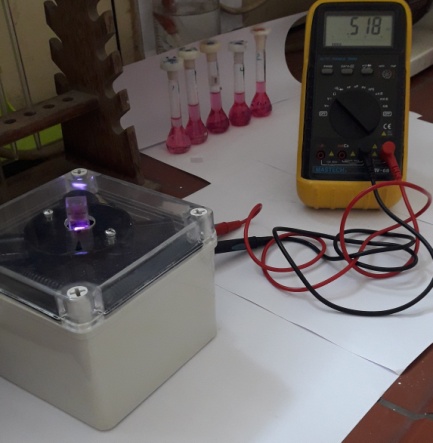  350 mg/L |  |
| 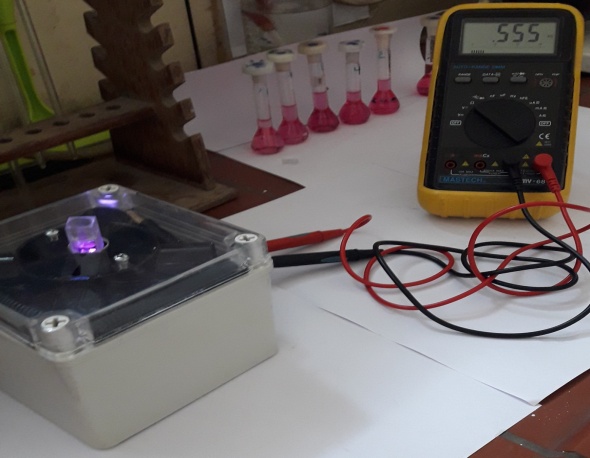  400 mg/L | | |

**Figure S1: Images showing the measurement of methyl red dye at concentrations of (100, 150, 200, 250, 300, 350, 400) mg/L using the new system.**

**Table S1: Resistance converted to absorbance for methyl red dye using a new LED-LDR PCM device**

| Dye Concentration  (mg/L) | Resistance value  (measured in KΩ) | Absorbance=log ($\frac{R_{sample}}{R_{blanck}}$) |
| --- | --- | --- |
| 100 | 0.364 | 0.862131 |
| 150 | 0.398 | 0.900913 |
| 200 | 0.43 | 0.934498 |
| 250 | 0.457 | 0.960946 |
| 300 | 0.495 | 0.995635 |
| 350 | 0.528 | 1.023664 |
| 400 | 0.565 | 1.053078 |
| Distilled water as Blank | 0.05 |  |

**Table S2: CaFRI tool evaluation for the determination of Methyl red dye using a new LED-LDR PCM device**

| **Parameter** | | **CaFRI** |
| --- | --- | --- |
| Energy | 1 - An energy reduction program or clean energy sources are adapted throughout the procedures | Yes |
|  | 2 - Total electrical power use of analytical instruments: | <0.1 KWh |
|  | 3 - Energy-intensive non-analytical equipemnts are essential: | No |
|  | 4 - Number of samples analyzed per hour: | 10-30 sampel/h |
| **CO₂** emission | 5 - The carbon footprint of the electrical power of analytical instruments is known | Yes |
|  | 6 - Emission factor: | <0.1 Kg CO2/KWh |
| Storage | 7 - Sample Storage: | No storage |
| Transportation | 8 - The sample has to be transported to an analytical Laboratory: | No |
|  | 9 - Distance between the sample field and the laboratory: | Not applicable |
|  | 10 - Number of samples transported in one shipment: | Not applicable |
|  | 11 - An ecofriendly vehicle is used in transportation: | Not applicable |
| Personnel | 12 - Number of personnel required for one sample analysis: | 1 person |
|  | 13 - Automation: | semiautomatic |
| Waste | 14 - Waste amount: | <10 mL |
|  | 15 - Waste disposal: | No waste disposal |
| Recycling | 16- Recycling | No recycle |
|  | 17 - Total number of pictograms: | ≤3 |
|  | 18 - Total amount of organic solvents per sample: | < 5 mL |
|  | 19 - Total amount of reagents per sample: | > 3 mL |
| Step 1: Calculating the energy consumption (kWh) of the lithium-ion battery | | Voltage (V): 3.7 V (from the battery).  Capacity (mAh): 9900 mAh, which is equivalent to 9.9 ampere-hours (Ah).  Energy (Wh)=Voltage × Capacity = 3.7 V × 9.9 Ah = 36.63 Wh.  Energy Consumption (kWh): 36.63 / 1000 = 0.03663 kWh. |
| Step 2: Determining the emission factor in Iraq | | Electricity generation in Iraq relies heavily on fossil fuels, leading to a high emission factor.  An approximate estimated value is 0.8 kg of CO2 per kilowatt-hour (0.8 kg CO2/kWh), which is a typical value for the Middle East region. |
| Step 3: Calculating the carbon footprint (kg CO2) for a single charge | | Carbon Footprint (kg CO2) = Energy Consumption (kWh) × Emission Factor (kg CO2/kWh).  Carbon Footprint = 0.03663 kWh × 0.8 kg CO2/kWh ≈ 0.0293 kg of CO2. |

**Table S3: MA tool evaluation for the determination of Methyl red dye using a new LED-LDR PCM device**

| **Parameter** | | **Score of**  **MA tool—Multi-color** |
| --- | --- | --- |
| 1- Where is the sample prepared? | ON- site | 2.38 |
| 2- Is the sample damaged during preparation? | No | 4.76 |
| 3- What extraction mode is used? | Microextraction (volume of cell 5 mL) | 3.57 |
| 4- What is the sample size? | < 10 mL (volume of cell 5 mL) | 4.76 |
| 5- How is the sample stored? | Room temperature | 4.76 |
| 6-Is derivatization required? | No derivatization need | 4.76 |
| 7-What are the hazards of the reagents used? | No hazardous reagent ( Methyl red dye dissolved in Distilled water) | 4.76 |
| 8-How much reagent is used per analysis? | Minimal (just Distilled water was used in this study) | 4.76 |
| 9-How many analytes are detected in a single run? | 1 analyte | 0.95 |
| 10-What is the sample throughput per hour? | ≥10 sample/h | 4.76 |
| 11-How many steps are involved in the method? | ≤3 steps | 4.76 |
| 12-What percentage of materials are sustainable? | 75% sustainable | 4.76 |
| 13-What is the cost per sample analysis? | 10-50 USD (24 $) | 2.38 |
| 14-What is the energy consumption per analysis? | Minimal (<0.1 KWh) (lithium-ion battery) | 4.76 |
| 15-Is the instrument automated? | Semi-automated | 3.14 |
| 16-Is the instrument miniaturized? | Partially miniaturized | 4.76 |
| 17-How is the waste treated? | Untreated | 0 |
| 18-What is the hazard level of waste produced? | Low hazard waste ( Methyl red dissolved in Distilled water) | 3.57 |
| 19- How much waste is produced per analysis? | Minimal <10 mL(volume of cell 5 mL) | 4.76 |
| 20-Are the procedures hermetically sealed? | Yes, fully sealed | 4.76 |
| 21-How noisy is the analytical process? | Silent operation (<40 dB) | 4.76 |
| 22-What type of analysis does the method provide? | Quantitative only | 7.5 |
| 23-How many analytes can be simultaneously determined? | Single analyte | 2.5 |
| 24-What analytical technique and instrumentation is used? | Simple/portable | 10 |
| 25-How many samples can be simultaneously treated? | 1 sample only | 2.5 |
| 26-What type of sample preparation is required? | On site prepration | 10 |
| 27-What is the total throughput in samples per hour? | ≥10 sample/h | 10 |
| 28-What type of reagents and materials are used? | Common (Distilled water) | 10 |
| 29- Is preconcentration required for the analysis? | No required | 10 |
| 30- What is the degree of automation? | Semi-automated | 7.5 |
| 31- What amount of sample is required? | ≤10 mL(volume of cell 5 mL) | 10 |
| 32-What is the repeatability (RSD%) of the method? | < 2% | 2.5 |
| 33-What is the intermediate precision of the method? | Not test | 0 |
| 34-What is the reproducibility of the method? | Not test | 0 |
| 35-What is the trueness (bias%) of the method? | <1% (compare with UV−Vis Spectrophotometer at 523 nm) | 10 |
| 36- What is the recovery and matrix effect performance? | >98% recovery | 7.5 |
| 37- What is the limit of quantification relative to expected levels? | <25% | 2.5 |
| 38- What is the working range (upper limit/LOQ ratio)? | >10 ×LQO  $\frac{400mg/L}{66.143 mg/L LQO of run 3}=6.05$ | 5 |
| 39-What is the linearity (R²) of the calibration curve? | >0.99 | 10 |
| 40-How many factors were tested for ruggedness/robustness? | No factor test | 0 |
| 41-How many interferents were tested for selectivity? | No interferent test | 0 |
| 42-Does the method use advanced sample preparation or instrumentation (e.g., SPME, LIS, DLLME, HRMS, MS/MS)? | Yes, high innovation | 10 |
| 43-Does it incorporate innovative data processing tools (AI, ML, in silico, blockchain, bioinformatics) and/or apply structured AQbD tools such as Design of Experiments (DoE), multivariate optimization, and statistical risk analysis? | Yes, Moderate innovation | 5 |
| 44-Does it consider White Analytical Chemistry principles or metrics (AGREE, BAGI, RAPI, ComplexGAPI, MoGAPI)? | Yes, high innovation | 10 |
| 45-Does it address guidance from relevant regulatory organizations or legal bodies? | Not addressed | 0 |
| 46-Does it use innovative reagents (e.g., MOFs, ILs, DES, 3D-printing, carbon dots, MIPs)? | Yes, high innovation (Distilled water more green solvent) | 10 |
| 47- Does it integrate miniaturized devices (portable systems, microfluidics, lab-on-chip, smartphones)? | Yes, high innovation | 10 |
| 48- Does it integrate automation (robotics, on-flow systems, automated prep or online injection)? | Yes, Moderate innovation | 5 |
| 49-Can the method be used across different fields (e.g., pharma, food, environment)? | Yes, high innovation | 10 |
| 50-Does it significantly improve LOD/LOQ values (nano or pico-level detection)? | No, conventional sensitivity | 0 |
| 51-Does it offer a new application, matrix, target analyte, or theoretical idea (e.g., hot topics)? | Yes, high innovation | 10 |

**Table S4: Reported methods details**

| **Reported methods** | **Ref.** |
| --- | --- |
| Sayyed Hossein Hashemi et al. used a Box-Behnken experimental design with seven factors at three levels to optimize the extraction efficiency (sample volume, pH, type and volume of eluent solvent, number of extraction and elution cycles, and the amount of sorbent).  A 10 mL sample solution containing methyl red was pretreated by adjusting the pH to 3.0 and adding 0.23 g of NaCl and 0.10% v/v of Triton X-114 as a surfactant. After the extraction, the dye was eluted with 300 µL of ethanol (repeated 9 times). The quantification was performed using a UNICO S200 Vis spectrophotometer at a wavelength of 485 nm. The method achieved a linear range of 3.0–300.0 μg/L and a limit of detection (LOD) of 0.50 μg/L. The mean recoveries for the dye in seawater samples were calculated to be 84.0–98.0%, with a mean relative standard deviation (RSD) of 2.5–6.7%. | 36 |
| Saeid Khodadoust and Mehrorang Ghaedi used dispersive liquid–liquid microextraction (DLLME) method for the determination of methyl red (MR) in water samples, followed by its analysis via UV–Vis spectrophotometry.  They used a Box-Behnken design (BBD) to optimize the extraction conditions, which included the volume of chloroform (as the extractant), the volume of ethanol (as the dispersant), pH, and ionic strength. The optimized conditions were determined to be 100 μL of chloroform, 1.3 mL of ethanol, a pH of 4, and 4% (w/v) NaCl.  For the extraction procedure, a 5.0 mL water sample, spiked with MR, was mixed with the solvent mixture. After centrifugation, the sedimented chloroform phase (approximately 70 μL) was collected. The solvent was evaporated, and the residue was redissolved in 2.00 mL of methanol for spectrophotometric measurement at 486 nm.  The method demonstrated a linear range of 0.005–100 mg/mL, with an R² value of 0.995. The limits of detection (LOD) and quantification (LOQ) were 0.001 mg/mL and 0.005 mg/mL, respectively, and the relative standard deviation (RSD) was less than 5%. | 37 |
| Masoud Shariati-Rad et al. developed a method for the removal, preconcentration, and spectrophotometric determination of methyl red using silica-coated magnetic nanoparticles (SiO₂-coated Fe₃O₄ MNPs).  The researchers used a central composite design to optimize factors such as pH, adsorbent dosage, and contact time. The optimal desorption conditions were a contact time of 3 minutes and 2.0 mL of 0.1 mol L⁻¹ NaOH, which resulted in a desorption efficiency of over 99%. The magnetic nanoparticles could be reused for two cycles with a removal efficiency of over 90%.  The quantification of the desorbed dye was done by spectrophotometry. The calibration curve was linear in the range of 0.025–0.250 ng/L, LOD of 0.174 ng/mL with a correlation coefficient (R2) of 0.9922. The method achieved a preconcentration factor of 50.  The method was applied to natural water samples, showing high accuracy (recoveries close to 100%) and precision (RSD < 1%). | 38 |
